# Estimating risk factor attributable burden – challenges and potential solutions when using the comparative risk assessment methodology

**DOI:** 10.1186/s13690-022-00900-8

**Published:** 2022-05-27

**Authors:** Dietrich Plass, Henk Hilderink, Heli Lehtomäki, Simon Øverland, Terje A. Eikemo, Taavi Lai, Vanessa Gorasso, Brecht Devleesschauwer

**Affiliations:** 1 German Environment Agency, Section Exposure Assessment and Environmental Health Indicators, Berlin, Germany; 2 National Institute for Public Health and the Environment (RIVM), Bilthoven, The Netherlands; 3 Finnish Institute for Health and Welfare (THL), Health Security, Environmental Health, Helsinki, Finland; 4 University of Eastern Finland (UEF), Faculty of Health Sciences, School of Pharmacy, Kuopio, Finland; 5 Section for Health Care Collaboration, Haukeland University Hospital, Bergen, Norway; 6 Centre for Global Health Inequalities Research (CHAIN), Department of Sociology and Political Science, Norwegian University of Science and Technology (NTNU), Trondheim, Norway; 7 Fourth View Consulting, Tallinn, Estonia; 8 Department of Epidemiology and Public Health, Sciensano, Brussels, Belgium; 9 Department of Translational Physiology, Infectiology and Public Health, Ghent University, Merelbeke, Belgium

**Keywords:** Burden of disease (BoD), Comparative risk assessment (CRA), Disability-adjusted life years (DALY), Health impact assessment (HIA), Population health

## Abstract

**Background:**

Burden of disease analyses quantify population health and provide comprehensive overviews of the health status of countries or specific population groups. The comparative risk assessment (CRA) methodology is commonly used to estimate the share of the burden attributable to risk factors. The aim of this paper is to identify and address some selected important challenges associated with CRA, illustrated by examples, and to discuss ways to handle them. Further, the main challenges are addressed and finally, similarities and differences between CRA and health impact assessments (HIA) are discussed, as these concepts are sometimes referred to synonymously but have distinctly different applications.

**Results:**

CRAs are very data demanding. One key element is the exposure-response relationship described e.g. by a mathematical function. Combining estimates to arrive at coherent functions is challenging due to the large variability in risk exposure definitions and data quality. Also, the uncertainty attached to this data is difficult to account for. Another key issue along the CRA-steps is to define a theoretical minimal risk exposure level for each risk factor. In some cases, this level is evident and self-explanatory (e.g., zero smoking), but often more difficult to define and justify (e.g., ideal consumption of whole grains). CRA combine all relevant information and allow to estimate population attributable fractions (PAFs) quantifying the proportion of disease burden attributable to exposure. Among many available formulae for PAFs, it is important to use the one that allows consistency between definitions, units of the exposure data, and the exposure response functions. When combined effects of different risk factors are of interest, the non-additive nature of PAFs and possible mediation effects need to be reflected. Further, as attributable burden is typically calculated based on current exposure and current health outcomes, the time dimensions of risk and outcomes may become inconsistent. Finally, the evidence of the association between exposure and outcome can be heterogeneous which needs to be considered when interpreting CRA results.

**Conclusions:**

The methodological challenges make transparent reporting of input and process data in CRA a necessary prerequisite. The evidence for causality between included risk-outcome pairs has to be well established to inform public health practice.

## Background

Burden of disease (BoD) analyses quantify the current level of population health and provide comprehensive overviews of the health status for countries or specific population groups. Since the first Global Burden of Disease (GBD) study published in the early 1990s, many countries have used BoD estimates to guide health policy decisions, as well as intervention and prevention measures to improve population health [1–4].

Public health systems can generally be adjusted to manage the identified burden from specific diseases and injuries flagged by BoD analyses on population scale. However, to reduce the disease burden and influence future health, it is essential to identify which risk factors are the key drivers of ill-health and death. One integral component of many BoD assessments is the attribution of disease burden to selected risk factors [5].

Risk-specific estimates unveil potentials to improve health and prevent disease and disability. The list of risk factors which in best case are modifiable through prevention and intervention measures is extensive. For instance, the GBD study framework has classified the included risks according to three major risk factor groups: a) metabolic, b) behavioral, and c) environmental and occupational risk factors [6]. The GBD 2019 study estimated that around 48% of the overall disease burden in 2019, measured in Disability-Adjusted Life Years (DALYs) can be attributed to the currently considered risk factors [6]. With about 831 million DALYs, the behavioral risk factors present the highest attributable burden, followed by 463 and 397 million DALYs for metabolic and the group of environmental and occupational risks, respectively [7]. Looking at the single risk factors globally, the highest attributable burden for 2019 was estimated for high systolic blood pressure (ca. 235 million DALYs), smoking (ca. 200 million DALYs) and high fasting plasma glucose (ca. 172 million DALY) [6]. The list of included risk factors is not exhaustive. However, missing data or insufficient evidence on the association between risk and outcome sometimes hamper the inclusion of further risk factors.

The aim of this paper is to identify and address some of the important challenges associated with the use of the comparative risk assessment (CRA) methodology, illustrated by selected examples and to discuss ways to handle them. We also use the example of social determinants to discuss difficulties that can arise when we want to expand the perspective and capture a broader set of risks in one common framework.

## Methods

We consulted and reviewed the main literature with respect to the CRA methodology and explain the main challenges when using this methodology with examples that should help the novice user to understand the concept and being aware of the pitfalls. We therefore screened the literature for risk-specific examples that present the challenges and possible solutions in the most educative way.

## Results

In BoD studies CRA methodology is commonly used to estimate the share of burden attributable to selected risk factors [8]. The general idea of the CRA is to compare a current harmful risk factor exposure level in the population against an alternative (or “counterfactual”) exposure situation where the selected risk factor is reduced to the so-called Theoretical Minimum Risk Exposure Level (TMREL). For example, the TMREL could correspond to zero *smoking*, the lowest observed concentration of *particulate matter in ambient air*, or sufficiently high levels of *whole grain consumption* [9].

**Fig. 1:**
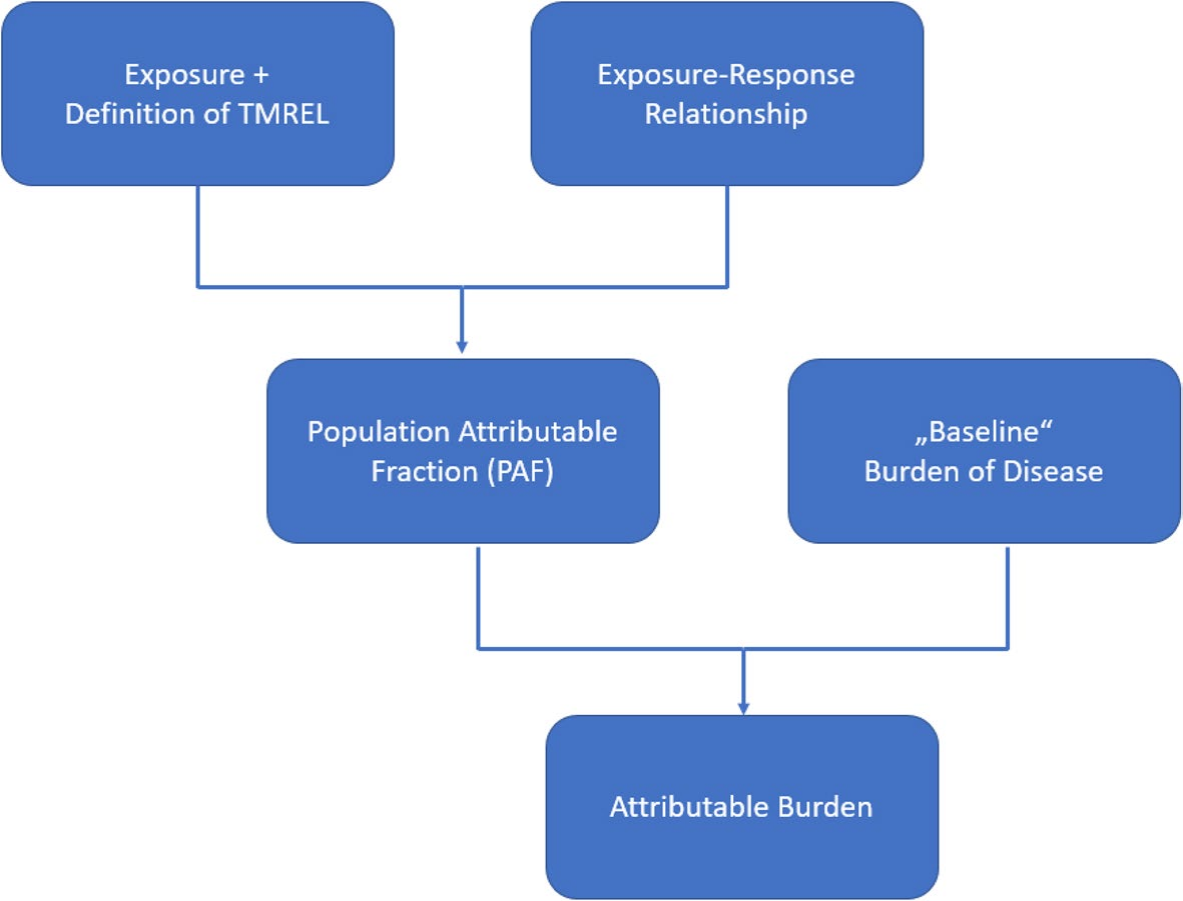
Flow diagram of the processes leading to attributable burden estimates (adopted from [10])

In general, the estimation of the fraction of disease attributable to a risk factor follows five consecutive steps, in the following described using illustrative examples (see also Fig. 1 for a simplified visual representation of the processes leading to estimates of attributable burden). Each of the steps comes with specific methodological and computational challenges, as exemplified. The different steps of the CRA are explained using the well-known example of smoking.

Step 1: definition of exposure (e.g., what kind of risk factor should be covered and how should the exposure be quantified?). In the case of smoking the exposure can be defined as actively smoking different types of tobacco (e.g. cigarette, pipe, e-cigarette). If one aims at a national burden of disease study one should consider all relevant tobacco types, because these can vary regionally all over the world.

Step 2: exposure assessment (e.g., how to measure or model the exposure of a population towards a risk factor?). In the case of smoking the exposure can hardly be measured adequately in the entire population. Therefore, representative samples can be used to estimate the overall smoking exposure of the population. Here, the exposure can be assessed by questionnaires, asking people about their smoking behavior. The smoking behavior can be quantified by cigarettes or packs smoked per day. An alternative could be to look for relevant markers (e.g. cotinine) of smoking in blood or urine samples, which is clearly more cost intensive as compared to the questionnaire.

Step 3: identification of risk-outcome-pairs (e.g., which health outcomes are causally related to the risk factor?)

In the case of smoking it is important to identify the relevant studies that quantify the increased risk of smoking for certain health outcomes. Here, for example prospective or retrospective cohort or case control studies can help to identify the health outcomes causally related to smoking.

Step 4: quantification of the association between outcome and risk (e.g., how does the risk increase with increasing levels of exposure to a risk factor?)

In the case of smoking the quantitative information on the association between outcome and risk can be extracted from the relevant epidemiological studies. The CRA allows to use relative risks (RR) or hazard ratios interpreted as relative risks. This information is necessary to later estimate the attributable disease burden by using the PAF.

Step 5: calculation of the attributable burden of disease (e.g., how to align definitions of relative risk and disease burden, how to account for combinations of risk factors?)

In the case of smoking it is necessary to first estimate the burden due to the relevant health outcomes. So, first the population level burden of disease estimates for e.g. lung cancer need to be quantified. This number is then multiplied by the PAF which represents a percental share of disease burden attributable to a certain risk factor.

Even though these five steps are generally universal for all risk factors, they pose different challenges for different risk factors when using the CRA framework. After having introduced the challenges, we apply these insights to the case of social determinants, an emerging topic within BoD literature. Finally, we discuss similarities and differences between CRA and HIA, as these concepts are often confused.

### Challenges and potential solutions

As many sets of estimates for risk factor attributable burden exist, it is of great value to know the pitfalls of such assessments. Especially for less experienced users the results of such assessments seem to be easy to understand and ad hoc comparable. It is however important to be aware of different components that can considerably impact the results. Here we present some selected important challenges of the CRA approach.

#### Identification of risk outcome pairs and the underlying causality

One key element of any CRA is the identification of the risk-outcome pairs for the considered risk factor(s). In general, a risk factor can be conceived as any exposure that increases the risk of developing a certain health outcome. In addition to the behavioural, metabolic, as well as occupational and environmental exposures as classified by the GBD study, a wide variety of other exposures may be considered as risk factors. For instance, infectious diseases may be considered as risk factors for ill health and mortality, as can genetic predispositions or socioeconomic status. Even health outcomes themselves (e.g. hypertension) can serve as risk factors for other health outcomes, adding to the complexity of the causal pathways.

Irrespective of the nature of the considered risk-outcome pair, an important first step is to establish causality. Indeed, the mere fact of finding the respective risk factor among patients, or finding a significant association in survey data, does not suffice to assume that the risk factor was the cause of the health outcome. The gold standard for concluding on causality is often considered to be a randomized controlled trial (RCT); in reality, however, it is not always possible, due to ethical or practical constraints, to perform RCTs for many risk-outcome pairs. Concluding on causality is therefore based on the strength of evidence that is brought by a variety of studies, each on its own not able to provide a definite answer. A commonly used framework to assess causality is the one proposed by Sir Austin Bradford-Hill [11]. For instance, the GBD study uses the World Cancer Research Fund criteria for “convincing or probable evidence” to include risk-outcome pairs [6]. These criteria are also influenced by the Bradford-Hill criteria. There is however ongoing discussion on the issue of causality when estimating the PAF and also the use of novel methodologies such as causal Bayesian frameworks is increasingly propagated [12, 13].

One example of a risk-outcome-pair from the GBD study is the causal association between ambient particulate matter and lung cancer. The evidence for the causal link between particulate matter and lung cancer was shown consistently in many studies. The evidence is convincing and was shown in epidemiological and toxicological assessments.

#### Definition of exposure and exposure assessment

A critical component in the CRA is the exposure assessment. The ways to estimate the exposure differ by risk factors and the data available in the relevant settings. Critical questions arise and need to be addressed when dealing with exposure assessment and the definition of exposure. How are humans exposed to the risk factor? Which pathways are relevant and which media should be analyzed to measure the appropriate exposure level? Using the example of air pollution and more specific particulate matter (PM) pollution it is already important to define the relevant size of the particles. Particulate matter can be split into more course particles (PM_10_), finer particles referred to as fine particulate matter (PM_2.5_), and the fraction of ultrafine particles (UFP) with aerodynamic diameter of 0.1 μm and smaller. Thus, defining the particle size which constitutes the health risk is vital for the CRA in this case. This choice sets the scene for all further steps and guides the estimation process.

#### Exposure misclassification

Even though exposure at population level is a necessary component of a CRA, the basis for such estimates are often epidemiologic studies where individual’s exposures to the risk factors of interest are captured. There are different ways to approximate the personal exposure of study participants with different levels of uncertainty. Depending on the selected risk factor the exposure can be detected by e.g. testing bodily fluids such as blood or urine for contaminants or their metabolites, using (standardized) questionnaires, measure the air quality surrounding the participant or using complicated models to approximate personal exposure. Using the example of air pollution several approaches can be used, which may yield varying results.

In the optimal case the study participants would wear personal measuring devices 24 hours and 7 days a week for several years. Based on this, longitudinal cohort studies may detect whether people developing or not developing health complaints were exposed to different levels of air pollution. Such designs would be costly, to a certain extent not realistic and particularly not available currently. It is also questionable whether using personal devices for several years is appropriate from an ethical point of view. Thus, other options need to be considered. In environmental epidemiology studies tackling air pollution, outdoor air pollutant concentrations are combined with the residential addresses of study participants. Common limitations of this approach are that only outdoor ambient air pollution is assessed, while the indoor-outdoor relationship of concentrations, as well as the participants’ spatial mobility or time activity patterns, are rarely accounted for. The first factor might have a diverse impact because studies show, that air quality levels for e.g. PM or nitrogen dioxide can vary between out- and indoors, due to different settings and especially different building characteristics e.g. ventilation rates and personal behavior e.g. cooking or smoking indoors [14]. Using only outdoor concentrations in the assessment of exposure can lead to an overestimation of the exposure [15]. Korhonen et al. 2019 estimated that taking into account infiltration in the residential outdoor based exposure model decreased the exposure estimates by up to 32% on average in the five European cities included in the study [15]. Considering infiltration might be even more important in future due to changes in building stock towards tighter buildings for increased energy efficiency, and consequently reduced infiltration of outdoor generated particles indoors [16]. Further, impacts of local indoor sources such as cooking or heating are not well captured in current epidemiologic studies. These increased concentrations however, represent temporary peaks and are probably less relevant for the long-term exposure.

The second argument especially holds when e.g. the home address and the working address have distinctly different exposure patterns (such as living in the country side but working in the city center). This impacts the overall long-term exposure, but such differentiations in exposure patterns are not yet well adjusted for in epidemiologic studies. Also, the exposure might change because of the relocation of participants throughout their life span, so that the current living address does not necessarily represent the actual exposure from the last couple of years. This can be considered in epidemiologic studies by asking the participants about their address history. It is sometimes argued that all factors may level out when combined. Nonetheless, as studies with long term follow-up using personal measurements are not available currently, and unlikely in the near future, approximating personal exposure by using area level exposure data is considered most appropriate. However, when interpreting results, such and other comparable limitations for each specific risk factor need to be considered carefully.

#### Identifying the exposure response relationships

Choosing an adequate exposure-response function (ERF) poses another key challenge in CRA and has a great impact on the resulting burden of disease. ERFs are also among the most significant sources of uncertainty in CRA because they provide the information about the strength of the association and about the size of risk increase at certain levels of exposure [17]. To define an ERF in most cases epidemiological studies which include effect measures, such as relative risks, odds ratios, or hazard ratios at certain exposure levels (e.g. concentration of particulate matter, number of cigarette pack-years) are mathematically combined to provide the information about the risk increase per unit increase of exposure to a certain risk factor. In an optimal case, the ERF is based on a systematic review and meta-analysis of recent studies. The units depend on the selected risk factor and can be e.g. concentration levels of particulate matter in μg/m^3^ or the number of cigarettes smoked per day or the amount of fruits eaten. A crucial prerequisite is that the definitions and units of the ERF and the exposure data need to match, otherwise incorrect results will be obtained.

The uncertainty associated with the ERF originates from the statistical uncertainty of a single ERF, but often also from the existence of multiple ERF for the same risk-outcome pair. Lehtomäki et al. 2020 compared two log-linear ERF with no threshold values applied and three sets of non-linear IER (Integrated Exposure Response) functions including thresholds for PM_2.5_ mortality in the five Nordic countries using same exposure and baseline health data. They found that the number of deaths attributable to PM_2.5_ in the Nordic area varied from 1800 to 18,000 depending on the chosen ERF. The Nordic area is especially sensitive to threshold values at the lower bound of the ERF because the concentrations are at relatively low levels [18].

#### Definition of the theoretical minimal risk exposure level

The definition of the TMREL is an often-underappreciated element in CRA. For some risk-outcome pairs, the choice is very evident. For instance, smoking unequivocally increases the risk of lung cancer, hence the TMREL would correspond to zero tobacco use. For other risk-outcome pairs, the zero-exposure level should not be considered as the TMREL, e.g. blood pressure where a level of systolic blood pressure at 120 mmHg is considered optimal, or the consumption of whole grains, where dose-response meta-analyses show that higher consumption levels are associated with decreasing risks.

In the context of air pollution, the identification of a TMREL is also one of the key questions in CRAs [19, 20]. For PM_2.5_ exposure and mortality, there are several ERFs presented and they vary regarding coefficients, shapes and possible thresholds. The WHO HRAPIE (Heath Risk of Air Pollution in Europe) working group for instance recommended quantifying the PM_2.5_ related mortality without a threshold (i.e. calculating the burden of disease for the whole exposure range) [21]. However, the integrated-exposure response functions (IER) and global exposure mortality model (GEMM) include theoretical thresholds under which burden of disease is not estimated due to the lack of knowledge about the shape of the ERF at the lowest exposure levels [22, 23]. Here the thresholds are defined as the lowest observed concentrations in the included cohort studies. It is also still under debate whether effects of natural particulate matter emissions such as sand storm related particulate matter, which has a different chemical composition should be considered in CRA. Nonetheless, defining the TMREL remains a crucial step and the uncertainty, as indicated by the relevant epidemiologic studies should be considered when interpreting the results. Meaningful, and when it comes to defining prevention and intervention measures, also feasible “lowest” risk level should be considered.

#### Calculation of population attributable fractions

Formally, the population attributable fraction (PAF) is defined as the proportion of cases for an outcome (e.g. lung cancer) of interest that can be attributed to an historical exposure to any given risk factor among the entire population (e.g. smoking):

$$PAF=\frac{I_{pop}-{I}_{unexposed}}{I_{pop}}$$

In CRA, e.g. the number of incident cases (I) (e.g. lung cancer) in the total (current) and unexposed (counterfactual) population, is obtained by combining information on the exposure distribution (e.g. how many people smoke certain amounts of cigarettes) and the relative risk linking exposure to outcome incidence:

$$PAF=\frac{\int P(x) RR(x)\mathrm{d}x-\int P^{\prime }(x) RR(x)\mathrm{d}x}{\int P(x) RR(x)\mathrm{d}x}$$

Where *P*(*x*) is the observed exposure distribution, *P* ’ (*x*) the counterfactual exposure distribtuion, and *RR*(*x*) the relative risk at a certain point on the ERF.

For a categorical exposure (e.g. weight status classified as normal weight, overweight, obesity), the continuous version of the PAF formula reduces to a discrete version:

$$PAF=\frac{\sum_{i=1}^n{P}_iR{R}_i-{\sum}_{i=1}^n{P}_i^{\prime }R{R}_i}{\sum_{i=1}^n{P}_iR{R}_i}$$

Where *P*_*i*_ is the observed prevalence of exposure class *i*, *P*’_*i*_ is the counterfactual prevalence of exposure class *i*, and *RR*_*i*_ the relative risk of exposure class *i* compared to the reference class.

When there are only two exposure classes (e.g. smoking classified as smoker vs non-smoker), the formula further reduces:

$$PAF=\frac{\left(P-{P}^{\prime}\right)\left( RR-1\right)}{P\left( RR-1\right)+1}$$

Often, one of the two exposure classes is considered the TMREL, hence *P* ’  = 1 (as in the smoker vs non-smoker example), then the formula further reduces to Levin’s formula:

$$PAF=\frac{P\left( RR-1\right)}{P\left( RR-1\right)+1}$$

Finally, if the exposure reflects an average population level exposure (e.g., air pollution), then the entire population is exposed to this risk, hence *P* = 1, and the formula reduces to its most simple form:

$$PAF=\frac{RR-1}{RR}$$

The different versions of the PAF formula thus reflect different definitions and units of the exposure and relative risk function. It is crucial to apply the correct formula to the available data, and to ensure that the definitions and units of the exposure data and relative risk functions are consistent.

#### Combined effects of risk factors

In a majority of CRA, attributable burden is estimated on the basis of the effect ascribed to a single risk factor, without considering possible interactions and combined effects of risk factors on population health. In reality, people are exposed to multiple risk factors or other protective or harmful health determinants that may or may not interact or accumulate towards health outcomes. Especially, in CRA of comprehensive burden of disease studies in which various risk factors are often considered, one has to account for these multiple exposure settings in order to correctly estimate the joint burden of different risk factors and not overestimate the population health impact. The considered determinants can have an influence on the same endpoints and the PAF should not just be casually added up without further processing. A simple sum would attribute too much impact to specific determinants, with the possibility to add up to more than 100% of the actual overall burden. For instance, ischemic heart disease is related to many different risk factors (e.g. dietary risks, smoking, air pollution) and its sum would almost reach 270% (see Table 1). Similarly, risk factors for lung cancer would add up to 133%. To correct for these factors, the PAF can be multiplicatively combined with the same endpoint ensuring that the value of the combined PAF does not exceed 1. This is done under the assumption that the effects of determinants occur independently considering the same endpoints. Though there is dependency among risk factors, for example smoking and alcohol occur in individuals almost double as much compared to independent occurrence [24], taking this into account when calculating a combined PAF requires far more data. The formula to calculate a combined PAF for a certain disease is therefore:

1 $${PAF}_{combi}=1-{{\prod}_i}\left(1-{PAF}_i\right)$$

Where the combined PAF is a result of the multiplication over the PAF for each risk factor *i*.

**Table 1:** Level 2 risk factors for ischemic heart disease, and tracheal, bronchus and lung cancer, European Union, 2019 (as % of DALYs) [7]

Risk factor:	Ischemic heart disease	Tracheal, bronchus, and lung cancer
Dietary risks	56%	4%
High systolic blood pressure	56%	
High LDL cholesterol	48%	
High fasting plasma glucose	29%	10%
Tobacco	27%	73%
High body-mass index	25%	
Air pollution	10%	9%
Low physical activity	6%	
Kidney dysfunction	12%	
Other environmental risks	2%	6%
Alcohol use	−2%	
Occupational risks		23%
Non-adjusted sum	269%	125%

In an optimal scenario the use of relative risks that are adjusted for other concurrent risk factors would probably yield the best estimates for the combined effects. Cohort studies and case-control-studies should increase the number of controlled confounders to deliver better risk estimates. Currently the use of univariate relative risks combined with a multiplicative adjustment of the PAF seems to be a pragmatic way to overcome the overestimation of risk factor attributable burden.

Mediation represents an additional challenge when generating estimates for the joint effect of risk factors, where the effect of one risk factor, partly or fully, goes through that of another risk factor. For instance, the GBD study assumes that the effect of low milk consumption entirely goes through the associated low calcium intake. Generating joint estimates of low milk and low calcium consumption would thus require corrections that go beyond the effect of the multiplicative model. The GBD study addresses this issue by incorporating mediation factors in the multiplicative model:

$${PAF}_{combi}=1-{\prod_{i}}\left(1-PAF_1{\prod_{m}}\left(1-MF_m\right)\right)$$

Where *MF*_*m*_ is the mediation factor for mediator *m* of risk factor *i*.

In the example of low milk and low calcium intake, the mediation factor for milk (calcium) is set to 1; as a result, the PAF for the joint effect equals the PAF of low calcium intake only. For other pairs, where the effect is not entirely mediated through one of the pair members, the mediation factor lies between 0 and 1.

#### Consistency in time dimension of risk and outcomes

It is likely that CRA is performed using the current exposure to a risk factor in a certain year to estimate the PAF and thus the attributable burden in that same year. Consequently, exposure and effect are captured at the same time, the year of analysis. However, in general, CRA is designed to attribute disease burden to past exposure. Having an effect from a risk factor immediately after exposure might be correct for acute adverse outcomes mostly connected to comparably high exposures. For long-term effects, such as those for smoking or air pollution, this assumption does not hold, as these risks work on longer time-frames and health effect result from chronic and cumulative exposures. Where possible CRA users should obtain not only the exposure to such risk factors in the year of interest but also estimate the historical cumulative exposure for example the average amount of cigarettes smoked during the life time of a person measured by using the indicator “pack-years” addressing the cumulative smoking history.

#### Heterogeneous evidence for the association between risks and outcomes

The CRA methodology used in the GBD 2019 study comprises a large set of risk factors with considerable variation in data availability and quality to inform the estimates. This also holds for other assessments where many risk factors are covered and when no transparent description of the input data sources is available. The missing transparency hampers a meaningful comparison of risk factors. At the end of most assessments, estimates of attributable disease burden are provided with a mean value of e.g. attributable DALYs, and in the best case accompanied by an uncertainty or confidence interval that quantifies the precision of the estimate. While the intervals indicate the uncertainties in the model parameters, it mostly remains unclear which component of the model led to these uncertainties. Especially, the evidence of the relative risk information can vary largely. For some risk-factors such as some single dietary risk-outcome-pairs in the GBD 2019 study, estimates come from randomized controlled trials. For other risks, such as particulate matter, risk estimates are from observational cohort studies. In the GBD 2019 study, risk estimates from various study designs are pooled to arrive at risk-outcome-pair specific effect estimates. While this improves the overall evidence compared to using single study estimates, it leaves ambiguity around the number and quality of the studies that provide input.

Also, some primary studies provide relative risk estimates by age and sex, while others do not. This can leave differences in risk sizes between age and sex undetected. CRA should preferably use stratified risk estimates where available to avoid under- or overestimation of attributable disease burden. As shown above, the impact of the RR, be it a single RR or RR combined in an ERF can be considerable and can lead to skewed comparisons. However, the granularity of risk estimates for risk-outcome pairs again is strongly linked to the stratification options provided by the underlying epidemiologic studies.

Another major challenge is that in many cases, the same relative risk estimate is used for both mortality and morbidity. Indisputably, these limitations are mostly related to the underlying epidemiologic studies fed into the model, and not to the CRA concept per se. However, the source of the RR estimates and its quality should be rated objectively and stated as a mandatory reporting component in CRA studies.

This heterogeneity in the evidence underlying the PAF and the burden estimates often leads to implicit assumptions and extrapolations. While this heterogeneity is inevitable, it is important to make these assumptions explicit, and to discuss possible limitations or develop alternative scenarios to quantify the associated uncertainties.

### Considering social determinants as risk factors in the CRA concept

In the available CRA estimates, also from the GBD-study, the focus remains on the classical risk factors from the four domains, metabolism, behaviour, occupational and environment. Increasingly, social determinants are recognized as important drivers of population health and health inequalities within and between population groups. Social determinants are therefore highly relevant for burden of disease studies, but arguably not yet considered adequately in CRAs. We therefore discuss the challenges when using CRA for estimating the burden attributable to social determinants.

Social determinants impact health through material, psychosocial, and cultural-behavioural pathways [25]. Socially patterned exposure to many of the risks across the three risk factor groups used in the GBD study illustrate the relevance of considering social inequality in CRA frameworks [26]. Beyond socially patterned exposure to behavioural risk factors, or material risk brought by absolute levels of poverty, low social position relative to others is also considered a risk factor on its own. These multidimensional links between social determinants and health outcomes pose several challenges when including social determinants in CRA, where the ultimate goal is to standardize assessments of risk factor impacts on population health. Some examples of such challenges are illustrated in the following, using “education” as an example of a social health determinant.

#### Identification of risk outcome pairs and the underlying causality

Available CRA assessments have so far focussed on biomedical risk models where it is assumed that risk-outcome pairs are universal throughout populations. As health inequalities emerge in the intersection between social structures, individual actions, and biological processes, there is a need to also include socially embedded and situated risk factors. However, this will pose challenges to the existing CRA modelling frameworks. For example, commonly used frameworks to assess causality, such as those offered by Bradford-Hill [11], and later frameworks have not been developed with social inequality as risk in mind. This should invite discussion on how such a causal framework could take shape.

#### Definition of the exposure and exposure assessment

What is the key feature of education that links it to health? Number of years of education? The level of education? The quality of education? Your own education or parental education? Attaining a diploma? Better grades than your peers? Is it the end sum of declarative knowledge or attaining general formative skills that later can be applied to life challenges? The literature is inconsistent on the matter, but in a CRA framework it should ideally be defined in a way that captures its essence, consistently and comparatively across regions and over time [27].

Inequalities in health between socio-economic groups are not restricted to differences between the most privileged and the most disadvantaged groups, but exist across social gradients [28–30]. This should favor a continuous measure of education. Number of years of education which has been shown to predict mortality and morbidity in a number of settings [31–33], although study results vary between subgroups and in mediation analyses [34]. Data availability at considerable geospatial resolutions lends further pragmatic support for using education as indicator for mapping social inequalities globally.

#### Identifying the exposure response relationships

Relative risk of disease given the exposure to a risk at a certain exposure level is required for calculating the PAF. The complex causal relationships between SES and outcomes complicate this, as also seen for other risk factors. Education (or other variables reflecting social inequality) can be conceptualized as a risk marker or higher level indicator that impact on a range of other exposures and risk behaviours more proximal to the health effects. If such hierarchical levels and mediation remain unaccounted for, large PAFs ascribed to education as a compound risk factor could be expected. It is yet not clear at what level social inequality should be included in a CRA framework and how models could partition its roles as both a risk marker and a risk factor, with burden estimates reflecting the conceptual choices correctly. This issue is related to the challenges described earlier, where mediation in causal pathways and combination of risks towards producing adverse health outcomes complicates attributing burden to single risk factors. Social inequality will in many respects operate through mediation and combinations of health behaviours that may result in diseases. Beyond this issue, the large time-span of relevance in the impact of social inequality is not unique, but challenging both for the primary studies needed to identify relative risk estimates, but also when identifying the right pairing between time of population exposure levels and the respective time of outcome identification.

#### Definition of the theoretical minimal risk exposure level

Compared to a unidirectional risk factor like smoking (0 smoking carries the minimum risk level), the TMREL for social inequality or a given indicator seems less certain. For education and in relative terms: Is no inequality in number of years of education carrying the minimal risk? Would that be uniform across outcomes and settings? And in absolute terms, is a given number of years of education likely to provide the lowest risk for adverse health outcomes? Again, as the CRA framework attempts to standardize risk over time and place, identifying a TMREL seems a conceptual and practical challenge.

In summary, social inequality in health is a core public health challenge and a risk factor for adverse health outcomes. Yet, more conceptual and empirical work is needed for social inequality to be included in CRA frameworks. How to assess and appraise causal roles of social inequality on health outcomes alongside other risk factors seems an important step. Many of the challenges will likely increase beyond using education as indicator, not at least due to sparser and less comparable data from existent observational studies. The ideal, but unfeasible approach would be to produce BoD-estimates stratified by social groups, as it currently done for sex and age groups. This would require stratified relative risks and exposure data, again resting on some of the same challenges with definitions, data and comparability.

### Comparative risk assessment versus health impact assessment

Both the CRA method and HIA are increasingly used. The CRA methodology is often confused with that of a HIA. They, however, serve different purposes and are based on different methodological frameworks. We therefore highlight the differences to help the novice users disentangle these concepts.

The main purpose of CRA is to generate comprehensive and comparable estimates of the current burden associated with risk factors and the historical exposure to these risk factors. On the other hand HIA aims to apply existing evidence and consider input from stakeholders to determine the potential effects of proposed policies, plans or interventions. Thus, a HIA is more a prospective tool that provides estimates of changes in disease burden related to changes in exposure resulting form given measures.

CRA provide theoretical estimates of the relative impact of risk factors. The estimates should probably serve more as “heuristics” than as “measured truths”. Furthermore, the definition of the TMREL merely reflects a hypothetical ideal world, without considering what is feasible or desirable. HIA, on the other hand, aims to support decision making by estimating the potential effect of realistic policy scenarios. Confusingly, however, the term HIA is sometimes also used for studies that aim to assess the health impact of risk factors.

Historically, the World Health Report (WHR) 2000 proposed BoD as a tool for monitoring health system performance in achieving health system goals of health outcome improvement and their equitable distribution [35]. The performance of health systems is of course dependent on the health system policies, programs and interventions implemented along their effectiveness as well as their intersectoral activities in ensuring positive outcomes of actions in other sectors as well [36]. Over the years, various concepts and tools such as effective coverage and WHO-CHOICE [37] have been used to provide direct links between health system interventions and both current and predicted future health system outcomes. A logical development in the same direction has been the extension of these BoD based approaches to measure health system performance also in achieving universal health coverage [38, 39].

In parallel, the HIA methodology has been promoted as a key instrument to safeguard public health [40, 41]. HIAs have been successfully and extensively used in urban planning, to assess the impacts of air pollution and transport [42–46] but also in other areas such as smoke free workplace policy and impact of health promotion campaigns [47].

Methodologically, HIA sets out to systematically judge the potential health effects of proposed policies, programs, or projects on population health and the distribution of those effects within a population by use of mixed-methods. HIA is thus intended to inform decision-makers by predicting the consequences in implementing different options, thereby enabling them to choose the option most beneficial for health and health equity [47]. HIA therefore ideally produces a practical set of recommendations for various policy options that can be incorporated into decision making process [46, 48]. As high proportion of HIAs focused on urban planning, traffic, agricultural policies and other areas outside the core focus of health systems, HIA is a significant Health in All Policies tool [49]. In this capacity it also helps to link progress in other sectors to achievement of the health-related SDGs while also providing evidence on effective interventions to improve population health outcomes and equity by action of other sectors [50–52].

In the increasingly connected world and with health systems relying ever more on community role and engagement in design and delivery health care services for strengthening person-centered health care systems HIA sets a good example. Namely, while there are numerous HIA frameworks, a common feature is systematic engagement of communities in scoping and assessing health impacts of the planned interventions [53]. HIA thus provide a model for how to create ‘knowledge spaces’ in which different perspectives and information can be brought around the table to create more democratic approaches to planning policies [54] while also providing a platform to bring BoD and CRA information to the communities thus, providing the link between high level policies and impact on members of the population.

## Conclusions and recommendations

Overall, the CRA methodology provides a set of standardized approaches to estimate the disease burden attributable to risk factors. Such estimates are vital to inform health policy decision makers on the key health determinants forming the current and also future health of populations. Knowing which risk factors are associated with the highest disease burden provides additional information for guiding prevention and intervention measures aiming at improving the health status of entire populations. However, key challenges are still to be tackled to see the whole picture. Only about one half of the overall disease burden is currently attributable to risk factors, and as shown in the paper, the position in the causal chain of events is not clear for all risk factors and it is likely that not all relevant risk factors and risk-outcome-pairs have been considered yet.

Given the different methodological challenges associated with CRA, it is indispensable to transparently report the input and process data of the models used in the CRA. It is also recommended to provide additional information on the evidence behind the associations of the selected risk-outcome-pairs to allow the reader to judge about the robustness of estimates and carefully compare estimates of different risks. Ad hoc comparisons especially of estimates provided by different CRA should be handled with caution and conclusions only drawn when having a transparent overview about the assumptions used. HIA can serve as potential addition to CRA as they provide information on how the current burden of disease will change in case of selected prevention and intervention options. This kind of forward-looking analyses can help decision makers to choose the options which are connected to highest benefits for the health of the population under study.

## Data Availability

The data that support the findings of this study are available from the Institute for Health Metrics and Evaluation and so are publicly available.

## Abbreviations

BoD: Burden of Disease
CRA: Comparative Risk Assessment
DALY: Disability-Adjusted Life Years
ERF: Exposure-Response Function
GBD: Global Burden of Disease study
HIA: Health Impact Assessment
PAF: Population Attributable Fraction
PM: Particulate matter
RR: Relative Risk
TMREL: Theoretical Minimum Risk Exposure Level
